# Datasets for spatial variation of O and H isotopes in waters and hair across South Korea

**DOI:** 10.1016/j.dib.2020.105666

**Published:** 2020-05-07

**Authors:** Mukesh Kumar Gautam, Byeong-Yeol Song, Woo-Jin Shin, Yeon-Sik Bong, Kwang-Sik Lee

**Affiliations:** a Division of Earth and Environmental Sciences, Korea Basic Science Institute, Ochang, Chungbuk 28119, Republic of Korea; b Biology Department, Medgar Evers College, City University of New York, New York, NY 11225, USA; c Chemical Analysis Division, National Forensic Service, Wonju 26460, Republic of Korea

**Keywords:** Oxygen and hydrogen isotopes, Isoscapes, Drinking water, Human hair, Geographic origin

## Abstract

The data presented here include the results of oxygen (δ^18^O) and hydrogen (δ^2^H) isotope analyses of water and human scalp hair samples collected from throughout the South Korea. The purpose of data collection was to generate isoscapes of oxygen and hydrogen isotopes for South Korea. To achieve the objective, we collected human scalp hair and three different types of water samples: groundwater, stream water and tap water. The data presented in the article are raw isotope data of water and hair samples in tabulated manner and interpolated isoscapes generated using those data. Further information related to the datasets and discussion about them can be found in the related research article entitled “Spatial variations in oxygen and hydrogen isotopes in waters and human hair across South Korea” [1].

Specifications table

**Table:** 

Subject	Earth and Environmental Sciences
Specific subject area	Isotope analysis and Isoscapes
Type of data	Table, Figure, Geospatial maps
How data were acquired	Isotope ratio mass spectrometers
Data format	Raw and analysed data
Parameters for data collection	All the isotopic ratios are reported in delta (δ) notation relative to Vienna Standard Mean Ocean Water (VSMOW), where δ (‰) = [(R_sample_/R_standard_) – 1] × 1000, and R = ^2^H/^1^H or ^18^O/^16^O. The analytical reproducibilities for δ^18^O and for δ^2^H were ±0.1‰ and ±1‰ for water samples, respectively, and 0.25‰ and below 2‰ for hair samples, respectively.
Description of data collection	56 groundwater, 130 stream water and 91 tap water samples, and 100 human scalp hair samples were collected across South Korea and analyzed for δ^18^O and δ^2^H. *d*-excess values were calculated using the equation: *d* = δ^2^H – (8 × δ^18^O) [2].
Data source location	Samples of different water types and human scalp hairs were from across South Korea from eight administratve units. Latitude and longitude (and GPS coordinates) for collected samples were given in the raw table presented in this article.
Data accessibility	With the article
Related research article	Gautam, M.K., Song, B.Y., Shin, W.J., Bong, Y.S., Lee, K.S., Spatial variations in oxygen and hydrogen isotopes in waters and human hair across South Korea [1]

## Value of the data

• The data will contribute to better understanding the geospatial variation in isotopes of water and hair on national scale and are an addition to the global isotope database
• Data are useful to other researchers who are interested in using water isotope for generating better global isoscapes and for those who use isotopes of drinking water and hair for provenance discrimination and in forensic analyses.
• The data will contribute to better understanding the application of oxygen and hydrogen isotopes as geographic tracer for agricultural products.
• The data can be used by other researchers who use isotope signatures of drinking water and human hairs for provenance discrimination and forensic analyses.

## 1 Data

**Fig 1:**
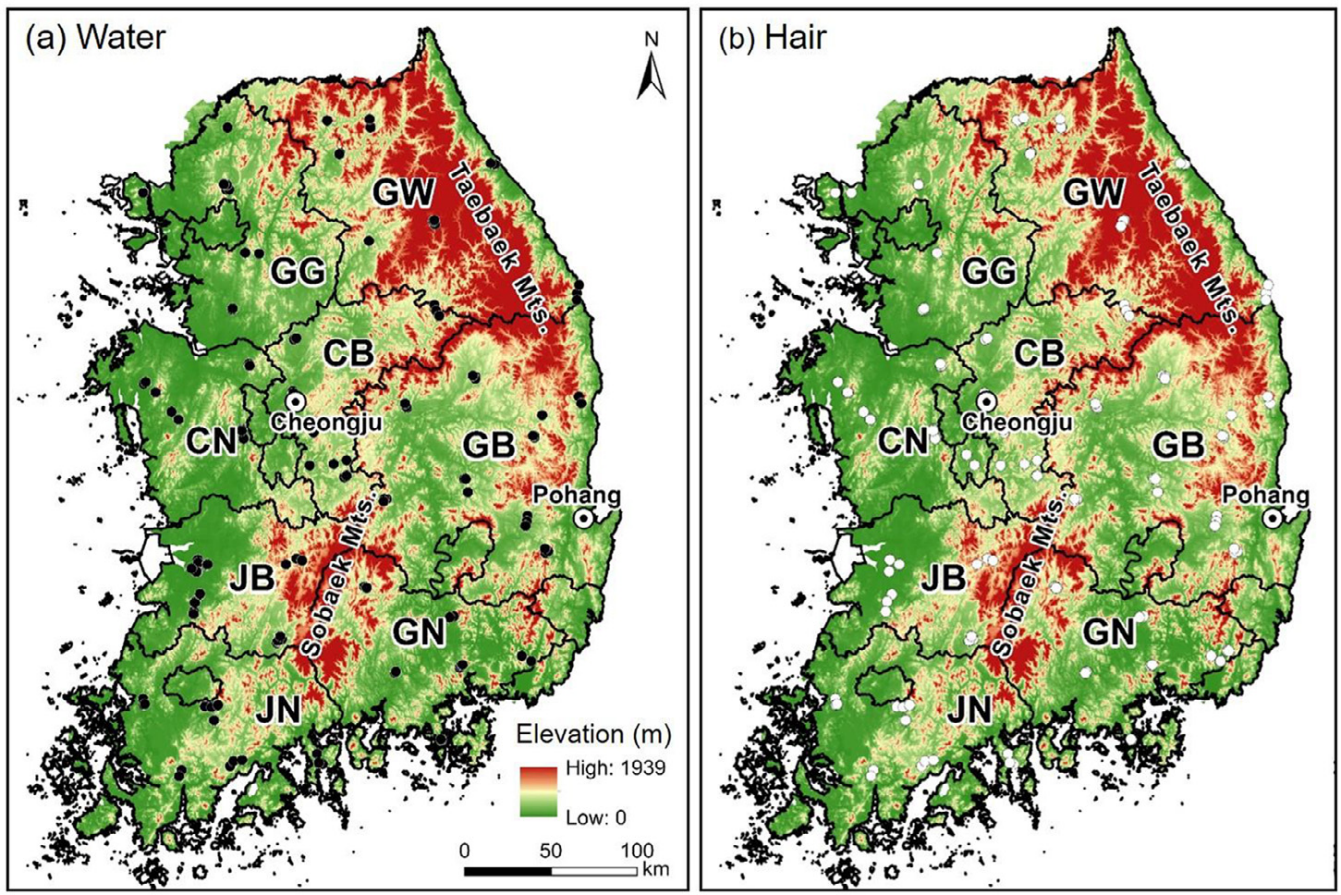
Spatial distribution of eight sampling provinces of groundwater, stream water and tap water, and human hair in the South Korea overlaid on elevation.Eight provinces are– Gangwon-do (GW), Gyeonggi-do (GG), Chungcheongbuk-do (CB), Chungcheongnam-do (CN), Jeolabuk-do (JB), Jeolanam-do (JN), Gyeongsangbuk-do (GB), Gyeongsangnam-do (GN), and two IAEA/WMO stations (Cheongju and Pohang).

**Fig. 2:**
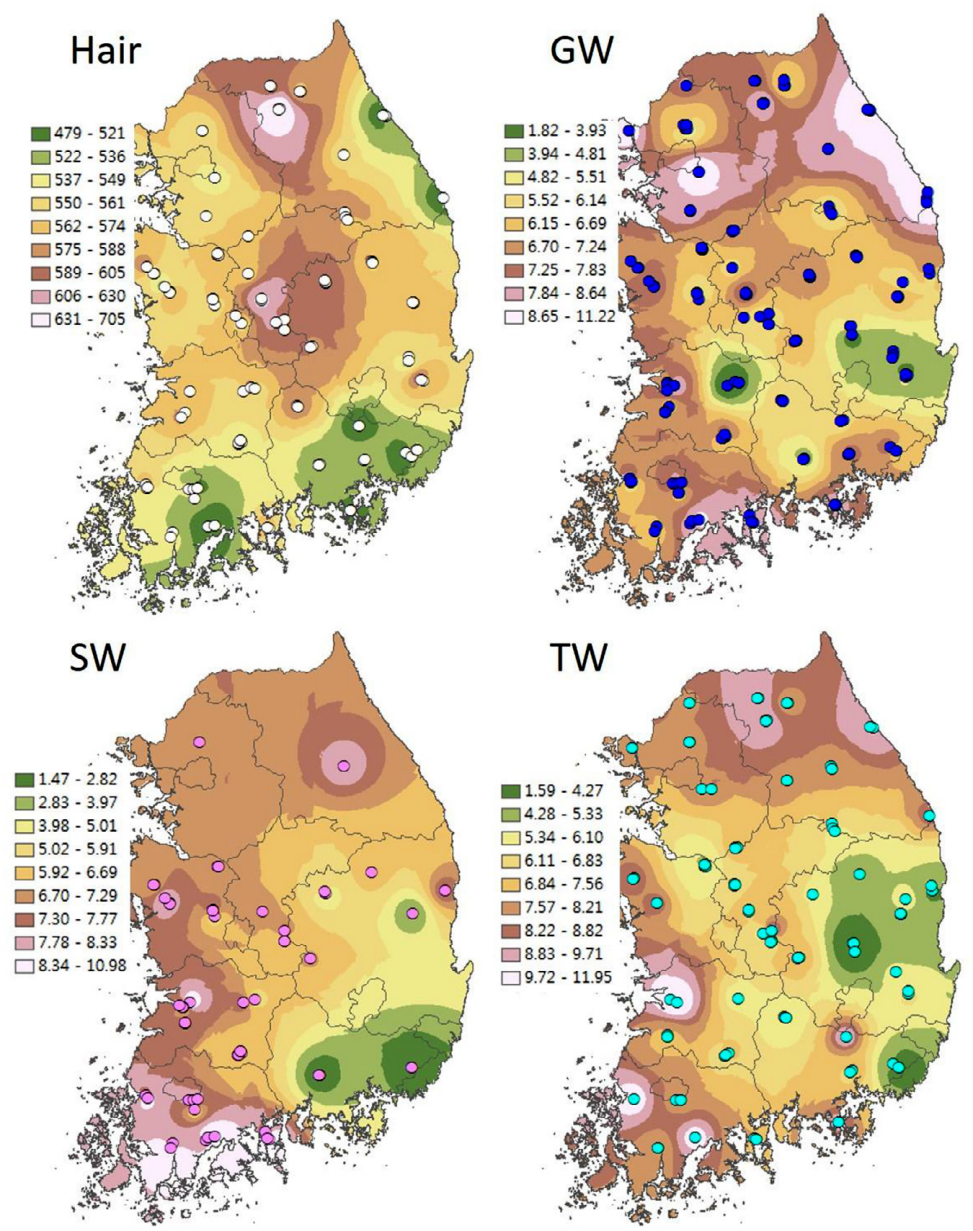
Spatial distribution of *d*-values of human hair and meteoric waters (GW, groundwater: SW, stream water; TW, tap water) across South Korea.

The data presented include oxygen (δ^18^O), hydrogen (δ^2^H) and *d*-excess values (*d* = δ^2^H – (8 × δ^18^O) [2] from three types of meteoric water (groundwater, stream water and tap water) and human scalp hair collected from across South Korea (Fig. 1). Table 1, Table 2, Table 3, Table 4 show raw δ^18^O, δ^2^H and *d*-excess data for groundwater, stream water, tap water and human scalp hair samples collected from different sites in South Korea. To understand the isotopic distribution on national scale, interpolated maps or isoscapes were developed, and spatial distribution of δ^18^O, δ^2^H and *d*-excess values were presented in Gautam et al. (in press) [1] and in the Fig. 2.

**Table 1:** Raw δ^18^O, δ^2^H and *d*-excess data for groundwater samples collected from different sites in South Korea.

Province	Sample ID	Latitude	Longitude	δ^18^O (‰)	δ^2^H (‰)	*d*-values (‰)
Chungcheongbuk-do	CB3-TG1	36.484	127.603	-8.40	-59.96	7.22
	CB4-TG1	36.338	127.816	-8.42	-59.87	7.48
	CB5-TG1	36.261	127.816	-8.48	-61.47	6.34
	CB5-TG3	36.257	127.816	-8.68	-63.89	5.57
Chungcheongnam-do	CN1-TG1	36.451	127.150	-7.88	-59.05	3.96
	CN1-TG2	36.499	127.138	-8.19	-55.06	10.46
	CN1-TG3	36.493	127.136	-8.77	-64.42	5.76
	CN2-TG1	36.830	127.184	-6.88	-50.05	4.96
	CN2-TG2	36.832	127.188	-8.35	-57.79	9.00
	CN3-TG1	36.550	126.728	-8.57	-60.17	8.39
	CN3-TG2	36.548	126.727	-7.41	-51.98	7.32
	CN3-TG3	36.589	126.682	-8.19	-56.81	8.67
	CN4-TG1	36.688	126.581	-7.81	-54.63	7.88
	CN4-TG2	36.689	126.576	-7.88	-57.58	5.44
Jeolabuk-do	JB1-TG1	35.794	127.424	-7.54	-52.12	8.20
	JB1-TG3	35.814	127.527	-7.72	-56.74	5.01
	JB2-TG1	35.404	127.400	-7.11	-51.00	5.87
	JB2-TG2	35.393	127.373	-8.74	-64.15	5.77
	JB2-TG2	35.393	127.370	-7.05	-50.68	5.72
	JB2-TG3	35.396	127.374	-9.13	-62.74	10.29
	JB2-TG3	35.396	127.375	-8.07	-59.31	5.24
	JB3-TG1	35.419	127.389	-8.23	-60.36	5.50
	JB3-TG3	35.419	127.389	-7.52	-54.12	6.05
	JB4-TG1	35.636	126.874	-8.02	-57.15	6.97
	JB4-TG1-1	35.639	126.874	-8.27	-57.80	8.40
	JB5-TG2	35.793	126.921	-9.33	-63.41	11.21
	JB5-TG3	35.756	126.856	-8.55	-66.36	2.06
	JB5-TG3-1	35.754	126.851	-9.06	-65.95	6.56
	JB5-TG3-2	35.754	126.851	-8.71	-60.61	9.10
	JB5-TG4	35.772	126.818	-9.53	-67.02	9.19
Jeolanam-do	JN1-TG1	35.088	126.516	-7.57	-53.50	7.10
	JN1-TG3	35.060	126.527	-7.45	-50.38	9.22
	JN2-TG1	35.049	126.911	-6.75	-46.60	7.37
	JN2-TG1-1	35.051	126.910	-7.14	-48.18	8.92
	JN2-TG2	35.047	126.952	-7.24	-50.63	7.28
	JN2-TG3	35.058	126.993	-7.16	-48.83	8.41
	JN3-TG1	34.718	126.764	-7.38	-52.19	6.83
	JN3-TG2	34.685	126.748	-7.16	-46.83	10.43
	JN3-TG3	34.979	126.968	-5.98	-43.33	4.51
	JN4-TG1	34.740	127.071	-6.63	-45.26	7.79
	JN4-TG2	34.766	127.093	-7.04	-47.68	8.67
	JN4-TG3	34.772	127.147	-7.45	-49.42	10.18
	JN5-TG1	34.806	127.622	-7.38	-52.22	6.79
	JN5-TG2	34.752	127.659	-6.99	-46.04	9.84
	JN5-TG3	34.759	127.633	-6.95	-46.50	9.12
Gyeongsangbuk-do	GB1-TG2	36.618	128.204	-8.34	-64.25	2.48
	GB1-TG3	36.633	128.196	-7.83	-55.63	7.05
	GB2-TG1	36.775	128.638	-8.40	-60.75	6.45
	GB3-TG2	36.123	128.053	-7.35	-51.92	6.86
	GB4-TG3	36.457	129.022	-8.48	-64.96	2.85
	GB8-TG2	36.621	129.339	-8.12	-57.18	7.77
Gyeonggi-do	GG1-TG3	37.779	127.014	-8.36	-59.84	7.03
Gyeongsangnam-do	GN2-TG2	35.231	128.126	-5.34	-42.38	0.34
	GN2-TG2-1	35.232	128.125	-6.59	-49.20	3.54
	GN4-TG3	35.280	128.989	-6.21	-48.25	1.47
Gangwon-do	GW6-TG1	37.588	128.398	-9.69	-69.33	8.19

**Table 2:** Raw δ^18^O, δ^2^H and *d*-excess data for stream water samples.

Province	Sample ID	Latitude	Longitude	δ^18^O (‰)	δ^2^H (‰)	*d-*value (‰)
Chungcheongbuk-do	CB1-S1	36.972	127.474	-7.21	-48.35	9.33
	CB1-S2	36.969	127.476	-8.13	-59.32	5.74
	CB1-S3	36.976	127.491	-8.17	-59.63	5.75
	CB2-S3	36.689	127.472	-7.47	-54.25	5.52
	CB3-S1	36.484	127.603	-8.54	-60.84	7.48
	CB3-S2	36.484	127.600	-8.55	-60.64	7.75
	CB3-S3	36.497	127.595	-8.75	-61.95	8.02
	CB4-S1	36.338	127.816	-8.39	-60.94	6.19
	CB4-S2	36.319	127.732	-8.44	-62.58	4.98
	CB4-S3	36.311	127.577	-8.25	-59.97	6.00
	CB5-S2	36.261	127.816	-8.26	-60.71	5.40
	CB5-S3	36.257	127.816	-8.35	-60.85	5.92
	CB6-S1	37.148	128.392	-8.88	-65.06	6.02
	CB6-S2	37.116	128.403	-8.46	-61.87	5.79
	CB6-S3	37.089	128.423	-8.97	-65.85	5.90
Chungcheongnam-do	CN1-S1	36.451	127.150	-7.66	-54.49	6.77
	CN1-S2	36.499	127.138	-7.50	-56.02	4.02
	CN1-S3	36.493	127.136	-8.20	-58.93	6.70
	CN2-S1	36.830	127.184	-7.57	-59.06	1.53
	CN2-S1-1	36.832	127.188	-7.68	-54.13	7.34
	CN2-S2	36.834	127.185	-8.10	-57.93	6.89
	CN2-S2-1	36.834	127.183	-7.65	-53.18	8.00
	CN2-S3	36.842	127.183	-8.26	-58.29	7.78
	CN2-S3-1	36.842	127.184	-7.53	-53.13	7.07
	CN3-S1	36.550	126.728	-8.06	-56.54	7.94
	CN3-S2	36.548	126.727	-7.39	-51.97	7.14
	CN3-S3	36.589	126.682	-7.74	-53.58	8.35
	CN4-S1	36.688	126.581	-8.05	-55.86	8.54
	CN4-S2	36.689	126.576	-8.42	-61.80	5.60
	CN4-S3	36.743	126.512	-8.43	-60.37	7.07
Jeolabuk-do	JB1-S1	35.794	127.424	-6.54	-50.47	1.84
	JB1-S2	35.825	127.492	-6.41	-47.98	3.32
	JB1-S3	35.814	127.527	-6.58	-48.87	3.77
	JB2-S1	35.404	127.400	-7.28	-53.71	4.51
	JB2-S2	35.393	127.373	-7.56	-56.13	4.33
	JB2-S2-1	35.391	127.372	-7.40	-53.32	5.86
	JB2-S3	35.396	127.374	-8.16	-57.38	7.92
	JB2-S3-1	35.393	127.370	-7.88	-56.31	6.73
	JB3-S1	35.419	127.389	-8.65	-57.26	11.92
	JB4-S1	35.636	126.874	-7.97	-58.72	5.07
	JB4-S2	35.599	126.843	-8.60	-61.36	7.46
	JB4-S2-1	35.593	126.841	-7.92	-56.22	7.11
	JB5-S1	35.817	126.859	-8.70	-63.74	5.86
	JB5-S1-1	35.801	126.861	-8.69	-62.92	6.63
	JB5-S2	35.793	126.921	-8.76	-63.32	6.79
	JB5-S2-1	35.790	126.916	-9.05	-60.23	12.14
	JB5-S3	35.756	126.856	-9.05	-64.25	8.15
Jeolanam-do	JN1-S1	35.088	126.516	-7.59	-50.40	10.31
	JN1-S2	35.057	126.519	-7.25	-52.91	5.07
	JN1-S3	35.060	126.527	-6.75	-49.40	4.61
	JN2-S1	35.049	126.911	-7.35	-51.40	7.40
	JN2-S2	35.047	126.952	-7.31	-48.35	10.12
	JN2-S3	35.058	126.993	-7.00	-48.83	7.15
	JN3-S1	34.718	126.764	-6.95	-48.90	6.72
	JN3-S2	34.685	126.748	-6.30	-44.48	5.95
	JN3-S3	34.979	126.968	-6.11	-41.72	7.19
	JN3-S3-1	34.977	126.969	-6.17	-44.50	4.85
	JN4-S1	34.740	127.071	-6.97	-47.60	8.19
	JN4-S2	34.766	127.093	-7.07	-49.27	7.28
	JN4-S3	34.772	127.147	-7.33	-48.75	9.92
	JN5-S1	34.806	127.622	-6.78	-45.04	9.17
	JN5-S2	34.752	127.659	-6.67	-45.21	8.15
	JN5-S3	34.759	127.633	-6.01	-40.42	7.70
Gyeongsangbuk-do	GB1-S1	36.610	128.208	-8.43	-59.95	7.45
	GB1-S2	36.618	128.204	-8.27	-59.81	6.38
	GB1-S3	36.633	128.196	-8.67	-61.72	7.66
	GB2-S1	36.775	128.638	-7.83	-56.75	5.91
	GB2-S2	36.770	128.655	-7.95	-57.38	6.21
	GB2-S3	36.759	128.652	-8.34	-59.96	6.73
	GB3-S1	36.134	128.073	-8.95	-65.75	5.88
	GB3-S2	36.123	128.053	-9.14	-65.74	7.36
	GB3-S3	36.136	128.057	-8.80	-63.64	6.77
	GB4-S1	36.447	129.032	-8.53	-62.70	5.51
	GB4-S2	36.456	129.032	-8.53	-60.18	8.05
	GB4-S3	36.457	129.022	-8.64	-62.90	6.18
	GB5-S1	36.039	128.979	-8.25	-61.86	4.14
	GB5-S2	36.010	128.974	-7.44	-54.91	4.63
	GB5-S3	35.987	128.964	-7.59	-56.60	4.09
	GB6-S1	36.236	128.574	-6.97	-53.82	1.92
	GB6-S2	36.236	128.584	-8.10	-54.39	10.40
	GB6-S3	36.167	128.598	-7.03	-54.34	1.90
	GB7-S1	35.842	129.092	-8.53	-60.45	7.77
	GB7-S2	35.852	129.109	-7.56	-57.38	3.12
	GB7-S3	35.866	129.088	-7.17	-55.86	1.51
	GB8-S1	36.564	129.084	-7.64	-56.04	5.05
	GB8-S2	36.621	129.339	-7.82	-57.24	5.33
	GB8-S3	36.658	129.324	-8.45	-59.75	7.85
Gyeonggi-do	GG1-S1	37.752	127.029	-7.74	-56.90	5.02
	GG1-S2	37.752	127.049	-7.09	-54.39	2.30
	GG1-S3	37.779	127.014	-8.98	-64.02	7.81
	GG2-S1	37.775	127.033	-8.51	-61.09	6.97
	GG2-S2	37.777	127.033	-8.36	-59.86	7.05
	GG2-S3	38.076	127.036	-8.81	-62.44	8.04
	GG3-S2	37.124	127.073	-8.40	-57.19	10.03
	GG3-S3	37.131	127.075	-8.01	-56.83	7.28
	GG4-S1	37.421	127.159	-8.58	-57.65	11.03
	GG4-S2	37.422	127.155	-8.80	-61.49	8.89
	GG4-S3	36.842	127.183	-8.74	-61.60	8.28
	GG5-S2	37.731	126.483	-8.03	-54.79	9.45
Gyeongsangnam-do	GN1-S1	35.678	127.925	-8.93	-64.97	6.47
	GN1-S2	35.677	127.934	-8.39	-62.25	4.84
	GN1-S3	35.672	127.942	-8.96	-65.50	6.15
	GN2-S2	35.231	128.126	-5.88	-44.75	2.27
	GN2-S3	35.226	128.119	-6.89	-48.40	6.71
	GN3-S1	35.260	128.540	-7.31	-52.45	6.04
	GN3-S2	35.263	128.549	-7.62	-54.10	6.89
	GN3-S3	35.265	128.551	-7.82	-53.47	9.13
	GN4-S1	35.308	128.929	-7.58	-52.14	8.48
	GN4-S2	35.280	128.987	-7.89	-56.84	6.25
	GN4-S3	35.280	128.989	-7.91	-57.49	5.80
	GN5-S1	35.519	128.498	-8.45	-61.06	6.54
	GN5-S2	35.514	128.471	-7.83	-57.77	4.89
	GN6-S1	34.876	128.413	-6.76	-45.93	8.12
	GN6-S2	34.878	128.408	-6.94	-47.29	8.25
Gangwon-do	GW1-S1	37.946	127.779	-9.08	-62.08	10.58
	GW1-S2	37.943	127.779	-8.92	-63.71	7.66
	GW1-S3	37.937	127.774	-8.91	-64.49	6.81
	GW2-S1	38.113	127.698	-10.02	-70.91	9.27
	GW2-S2	38.112	127.694	-9.58	-70.61	6.06
	GW3-S1	38.076	127.985	-9.02	-67.48	4.67
	GW3-S2	38.076	127.981	-9.38	-67.92	7.16
	GW3-S3	38.119	127.975	-8.72	-63.78	5.95
	GW5-S1	37.876	128.804	-8.99	-61.13	10.81
	GW5-S2	37.878	128.797	-8.04	-56.12	8.17
	GW5-S3	37.883	128.773	-8.14	-55.27	9.83
	GW6-S1	37.588	128.398	-9.49	-67.21	8.74
	GW6-S2	37.585	128.390	-9.43	-66.89	8.53
	GW7-S1	37.197	129.325	-8.07	-54.98	9.58
	GW7-S2	37.241	129.339	-7.87	-53.33	9.64
	GW7-S3	37.162	129.324	-8.63	-59.28	9.74

**Table 3:** Raw δ^18^O, δ^2^H and *d*-excess data for tap water samples.

Province	Sample ID	Latitude	Longitude	δ^18^O (‰)	δ^2^H (‰)	*d-*values (‰)
Chungcheongbuk-do	CB1-T1	36.972	127.474	-9.55	-68.26	8.14
	CB1-T2	36.261	127.816	-8.62	-63.28	5.66
	CB1-T3	36.976	127.491	-9.59	-67.89	8.80
	CB2-T1	36.700	127.467	-8.33	-59.71	6.93
	CB2-T2	36.683	127.467	-8.45	-60.97	6.65
	CB2-T3	36.689	127.472	-8.19	-59.02	6.51
	CB3-T2	36.484	127.600	-8.64	-63.50	5.62
	CB3-T3	36.497	127.595	-8.75	-61.38	8.61
	CB4-T2	36.319	127.732	-8.37	-62.37	4.62
	CB4-T3	35.383	127.367	-8.74	-60.69	9.23
	CB5-T2	36.261	127.816	-8.61	-62.21	6.69
	CB6-T1	37.148	128.392	-9.19	-65.60	7.93
	CB6-T2	37.116	128.403	-9.53	-70.17	6.07
	CB6-T3	37.089	128.423	-9.32	-69.63	4.96
Chungcheongnam-do	CN1-T1	36.969	127.476	-8.62	-63.28	5.65
	CN1-T2	35.533	126.833	-8.71	-64.41	5.30
	CN1-T3	35.383	127.367	-8.72	-64.84	4.89
	CN2-T1	36.830	127.184	-8.03	-59.08	5.18
	CN2-T1-1	36.830	127.184	-8.25	-57.95	8.06
	CN2-T2	36.832	127.188	-8.23	-60.24	5.62
	CN2-T3	36.842	127.183	-8.74	-64.47	5.47
	CN2-T3-1	36.842	127.183	-8.51	-64.48	3.61
	CN2-T3-2	36.842	127.183	-8.65	-64.96	4.23
	CN3-T1	36.550	126.728	-7.48	-51.09	8.71
	CN4-T3	36.743	126.512	-7.51	-51.24	8.85
	CN4-T4	36.733	126.500	-7.42	-51.07	8.33
Jeolabuk-do	JB1-T2	35.825	127.492	-8.03	-58.78	5.44
	JB2-T1	35.404	127.400	-8.15	-57.19	8.00
	JB2-T2	35.393	127.373	-8.20	-58.44	7.13
	JB4-T2	35.546	126.835	-9.64	-68.05	9.07
	JB4-T3	35.533	126.833	-8.42	-60.15	7.21
	JB5-T1	35.817	126.859	-9.05	-61.71	10.70
	JB5-T2	35.793	126.921	-9.12	-60.94	11.99
Jeolanam-do	JN1-T2	35.057	126.519	-7.70	-50.80	10.78
	JN2-T1	35.049	126.911	-7.50	-51.85	8.15
	JN2-T2	35.047	126.952	-7.87	-55.13	7.86
	JN3-T2	34.685	126.748	-7.27	-50.66	7.49
	JN3-T2-1	34.685	126.748	-6.44	-45.56	6.00
	JN4-T2	34.766	127.093	-6.97	-45.20	10.56
	JN5-T2	34.752	127.659	-7.19	-49.55	7.96
	JN5-T3	34.759	127.633	-7.66	-52.53	8.78
Gyeongsangbuk-do	GB1-T1	36.610	128.208	-8.31	-60.56	5.91
	GB2-T3	36.759	128.652	-8.06	-60.04	4.44
	GB3-T1	36.134	128.073	-9.03	-65.00	7.28
	GB3-T2	36.123	128.053	-9.18	-66.27	7.15
	GB3-T3	36.136	128.058	-7.33	-50.79	7.85
	GB4-T1	36.447	129.033	-8.75	-64.14	5.87
	GB4-T1-1	36.447	129.032	-8.79	-66.20	4.09
	GB4-T2	36.456	129.032	-8.76	-64.37	5.73
	GB5-T2	36.010	128.974	-8.66	-63.73	5.52
	GB6-T1	36.236	128.574	-7.61	-58.94	1.93
	GB6-T2	36.236	128.584	-7.74	-58.30	3.66
	GB6-T3	36.167	128.598	-7.68	-58.39	3.03
	GB7-T1	35.842	129.092	-8.57	-61.53	7.05
	GB7-T2	36.250	127.800	-8.48	-61.90	5.95
	GB7-T3	35.866	129.088	-8.44	-62.46	5.06
	GB8-T2	36.621	129.339	-7.47	-56.87	2.87
	GB8-T2-1	36.564	129.084	-7.95	-57.55	6.06
	GB8-T3	36.658	129.324	-8.09	-58.60	6.08
Gyeonggi-do	GG2-T1	37.775	127.033	-8.88	-63.36	7.69
	GG2-T2	37.777	127.033	-8.80	-62.08	8.32
	GG2-T3	38.076	127.036	-8.54	-60.25	8.04
	GG4-T1	37.421	127.159	-9.22	-65.87	7.92
	GG4-T3	37.418	127.248	-9.20	-65.26	8.36
	GG5-T1	37.733	126.483	-8.98	-63.82	8.06
Gyeongsangnam-do	GN1-T1	35.678	127.925	-9.23	-67.03	6.79
	GN1-T2	35.677	127.934	-8.40	-64.01	3.16
	GN1-T3	35.672	127.942	-8.71	-63.20	6.46
	GN1-T4	35.672	127.942	-8.84	-64.65	6.09
	GN2-T3	35.265	128.551	-9.46	-66.82	8.86
	GN3-T1	35.250	128.533	-7.21	-52.22	5.48
	GN4-T1	35.308	128.929	-7.74	-56.59	5.34
	GN5-T1	35.519	128.498	-8.80	-60.55	9.82
	GN5-T2	34.752	127.659	-6.98	-50.70	5.13
	GN6-T1	34.876	128.413	-9.08	-63.48	9.12
	GN6-T3	35.280	128.989	-6.21	-48.2	1.47
Gangwon-do	GW1-T1	37.946	127.779	-9.33	-66.92	7.70
	GW1-T2	37.943	127.779	-9.34	-65.80	8.90
	GW1-T3	37.937	127.774	-9.79	-67.31	10.98
	GW2-T1	38.113	127.698	-9.79	-68.67	9.62
	GW2-T2	38.112	127.694	-9.75	-68.48	9.51
	GW3-T1	38.076	127.985	-8.70	-63.90	5.69
	GW3-T2	38.076	127.981	-9.62	-67.29	9.66
	GW4-T1	37.483	127.967	-8.91	-63.48	7.80
	GW4-T2	37.483	127.967	-8.93	-63.48	7.98
	GW5-T1	37.876	128.804	-9.24	-64.38	9.52
	GW5-T3	37.883	128.773	-9.22	-63.89	9.85
	GW6-T2	37.585	128.390	-10.28	-74.67	7.53
	GW6-T3	37.567	128.400	-9.67	-69.31	8.07
	GW7-T1	37.197	129.325	-8.05	-55.86	8.56
	GW7-T2	36.338	127.816	-8.42	-56.37	10.95

**Table 4:** Raw δ^18^O, δ^2^H and *d*-excess data for human scalp hair samples.

Province	Sample ID	Latitude	Longitude	δ^18^O (‰)	δ^2^H (‰)	*d*-values (‰)
Chungcheongbuk-do	CB1-H1	36.972	127.474	10.92	-70.18	-157.5
	CB2-H1	35.825	127.492	10.41	-77.32	-160.6
	CB2-H2	35.825	127.492	10.68	-76.48	-161.9
	CB3-H1	36.689	127.472	9.90	-68.43	-147.6
	CB3-H3	36.484	127.600	9.73	-81.10	-158.9
	CB4-H1	36.497	127.595	10.43	-76.68	-160.1
	CB4-H2	36.338	127.816	10.32	-70.78	-153.3
	CB4-H3	36.319	127.732	10.63	-77.28	-162.3
	CB5-H2	36.261	127.816	10.88	-67.70	-154.7
	CB6-H1	36.257	127.816	9.11	-77.58	-150.5
	CB6-H2	37.148	128.392	10.52	-72.68	-156.8
	CB6-H3	37.116	128.403	10.42	-73.24	-156.6
Chungcheongnam-do	CN1-H1-1	37.089	128.423	10.70	-67.35	-153.0
	CN1-H2	36.451	127.150	10.50	-65.93	-149.9
	CN1-H3	36.499	127.138	11.26	-73.55	-163.6
	CN2-H1	36.493	127.136	10.86	-64.71	-151.6
	CN2-H1-1	36.830	127.184	11.02	-69.15	-157.3
	CN2-H2	36.830	127.184	10.77	-68.10	-154.3
	CN2-H3	36.832	127.188	10.11	-76.17	-157.1
	CN3-H1	36.842	127.183	11.40	-64.07	-155.3
	CN3-H2	36.550	126.728	11.39	-65.09	-156.2
	CN4-H2	36.688	126.581	10.84	-67.28	-154.0
	CN4-H3	36.689	126.576	10.48	-66.12	-150.0
	CN4-H4	36.743	126.512	10.26	-74.21	-156.3
	CN6-H1	36.313	127.407	11.46	-66.32	-158.0
Jeolabuk-do	JB1-H1-1	36.368	127.355	11.04	-71.20	-159.5
	JB1-H2	35.794	127.424	10.01	-66.01	-146.1
	JB1-H3	35.825	127.492	10.13	-69.50	-150.5
	JB2-H1	35.814	127.527	10.72	-63.96	-149.7
	JB2-H2	35.404	127.400	10.65	-66.37	-151.6
	JB2-H3	35.393	127.373	10.78	-73.29	-159.5
	JB3-H1-1	35.396	127.374	10.70	-62.74	-148.3
	JB3-H2-1	35.419	127.389	10.36	-64.27	-147.2
	JB4-H1	35.419	127.389	9.97	-67.52	-147.3
	JB4-H2	35.636	126.874	10.42	-70.33	-153.7
	JB4-H3	35.599	126.843	10.53	-70.89	-155.1
	JB5-H3	35.793	126.921	10.78	-69.71	-156.0
Jeolanam-do	JN1-H2	35.088	126.516	11.55	-74.23	-166.6
	JN1-H3	35.057	126.519	10.89	-61.27	-148.4
	JN2-H1	35.060	126.527	11.14	-68.29	-157.4
	JN2-H2	35.049	126.911	10.68	-65.48	-150.9
	JN2-H3	35.047	126.952	11.12	-69.22	-158.2
	JN3-H1	35.058	126.993	11.21	-59.57	-149.3
	JN3-H2	34.718	126.764	10.97	-64.88	-152.6
	JN3-H3	34.685	126.748	11.00	-67.45	-155.5
	JN4-H1-1	34.979	126.968	10.46	-65.46	-149.1
	JN4-H3	34.766	127.093	11.39	-58.40	-149.5
	JN5-H1	34.772	127.147	11.47	-61.06	-152.8
	JN5-H3	34.752	127.659	10.78	-67.81	-154.1
Gyeongsangbuk-do	GB1-H1	34.759	127.633	10.45	-71.22	-154.8
	GB1-H2	36.610	128.208	10.68	-72.34	-157.8
	GB1-H3	36.618	128.204	11.34	-72.31	-163.0
	GB2-H1	36.633	128.196	9.64	-76.09	-153.2
	GB2-H2	36.775	128.638	9.74	-73.31	-151.2
	GB2-H3	36.770	128.655	9.24	-70.83	-144.8
	GB3-H1	36.759	128.652	10.12	-64.60	-145.6
	GB3-H2	36.134	128.073	10.18	-71.32	-152.8
	GB3-H3	36.123	128.053	10.16	-72.76	-154.0
	GB4-H2	36.447	129.032	9.73	-72.20	-150.0
	GB4-H3	36.456	129.032	11.27	-66.79	-157.0
	GB5-H1	36.457	129.022	11.18	-68.59	-158.0
	GB5-H2	36.039	128.979	11.47	-70.44	-162.2
	GB5-H3	36.010	128.974	11.23	-62.36	-152.2
	GB7-H3	35.852	129.109	10.44	-64.60	-148.1
	GB8-H1	35.866	129.088	9.30	-78.69	-153.1
Gyeonggi-do	GG2-H2	37.775	127.033	10.45	-70.38	-154.0
	GG2-H3	37.777	127.033	10.03	-65.04	-145.3
	GG4-H1	37.131	127.075	10.88	-67.96	-155.0
	GG4-H1-1	37.421	127.159	11.04	-65.86	-154.2
	GG4-H3	37.421	127.159	10.15	-67.13	-148.3
Gyeongsangnam-do	GN1-H2	35.678	127.925	10.93	-74.04	-161.5
	GN1-H3	35.677	127.934	10.75	-68.97	-155.0
	GN2-H1	35.226	128.119	11.42	-64.31	-155.7
	GN2-H2	35.232	128.124	10.43	-61.74	-145.2
	GN2-H3	35.231	128.126	10.22	-68.34	-150.1
	GN3-H1	35.226	128.119	11.08	-65.27	-153.9
	GN3-H2	35.260	128.540	10.24	-65.77	-147.7
	GN3-H3	35.263	128.549	9.74	-65.72	-143.6
	GN4-H2	35.308	128.929	11.13	-59.75	-148.8
	GN4-H3	35.280	128.987	10.78	-63.68	-149.9
	GN5-H2	35.519	128.498	12.24	-60.63	-158.6
	GN6-H3	34.878	128.408	11.02	-62.92	-151.1
	GN7-H2	35.333	129.035	11.27	-67.87	-158.0
	GN7-H3	35.333	129.043	11.30	-64.55	-155.0
Gangwon-do	GW1-H1	37.946	127.779	10.94	-88.77	-176.3
	GW1-H2	37.943	127.779	10.31	-79.84	-162.3
	GW1-H3	37.937	127.774	10.33	-79.41	-162.1
	GW2-H1	38.113	127.698	10.11	-67.36	-148.2
	GW2-H3	38.115	127.690	11.34	-76.11	-166.8
	GW3-H1	38.072	127.982	9.90	-77.13	-156.3
	GW3-H2	38.076	127.985	10.10	-67.54	-148.3
	GW3-H3	38.076	127.981	9.86	-65.48	-144.4
	GW4-H1	36.550	126.728	10.34	-70.13	-152.9
	GW4-H2	36.548	126.727	10.59	-77.63	-162.4
	GW4-H3	36.589	126.682	11.30	-59.72	-150.1
	GW5-H1	37.876	128.804	9.99	-65.44	-145.4
	GW5-H2	37.878	128.797	10.34	-65.75	-148.5
	GW5-H3	37.883	128.773	10.20	-58.65	-140.3
	GW6-H1	37.588	128.398	10.35	-67.63	-150.4
	GW7-H2	37.241	129.339	10.80	-61.94	-148.3

## 2 Experimental design, materials, and methods

### 2.1 Study location

Our sampling sites for meteoric waters (groundwater, stream water and tap water) and hair samples were located across the country, encompassing all the eight administrative provinces of South Korea (Fig. 1).

### 2.2 Water samples

In September–October 2010, 56 groundwater, 130 stream water and 91 tap water samples were collected across South Korea, and their GPS coordinates and other metadata were recorded. Sampling emphasized obtaining nation-wide high-resolution datasets for the construction of reliable interpolated isoscapes. For successful sampling that incorporated the maximum variability, the campaign was carried out in urban, suburban and rural areas. Groundwater samples were obtained from local houses in person (offered by residents) and from automated bore wells. Prior to sample collection, water was allowed to flow out for 10 to 15 min to remove factors that might cause fractionation (e.g., water trapped in the pipes since the last use of the well) and to obtain well-mixed groundwater samples. When possible, we collected tap water (municipal water supply) samples from the same locations where groundwater sampling was conducted. Samples were collected only from in-use cold water taps, and the tap was run for one min prior to sample collection. Where feasible, the surface water and stream water sampling locations were chosen based on proximity to groundwater and tap water sampling locations. To remove the possibility of evaporative enrichment, samples were collected only from the flowing sections of streams. All water samples were filtered after collection through a 0.45-μm PVDP Millipore® syringe filter (Millipore Corporation, Billerica, USA) into 60-mL glass vials. Vials were filled to the neck and closed with rubber-lined caps to avoid exchange with the atmosphere. The vials were immediately stored in portable iceboxes with re-usable ice packs (to reduce photodegradation) after collection and transported to the laboratory. In the laboratory, the vials were stored at 4°C until isotopic analyses.

### 2.3 Human scalp hair samples

Human scalp hairs were sampled from different locations (barbershops) across South Korea (n = 100). Hair samples discarded at the barbershops were collected. The length was not taken into consideration, but it was usually 4-6 cm long and there is a weak possibility that a hair will mix with various lengths of hair. Only undyed hair samples were collected for the study. To ensure that the isotopic signature of hair samples reflected the geographic location of sampling regions, hair samples were obtained from local residents, when possible, who lived in close proximity to the barbershop. Care was taken to select barbershops near the water sampling sites. With this approach, we can assume that the individuals whose hair was collected had access to local water sources. In addition, three sets of discarded hair clippings were collected in the suburban towns to increase the probability that the hair samples were associated with local residents.

### 2.4 Stable isotope analyses

The oxygen and hydrogen isotope ratios of water and hair samples were measured using Optima isotope ratio mass spectrometer (VG Isotech, Middlewich, UK) and dual inlet Isoprime isotope ratio mass spectrometer equipped with an on-line Euro PyrOH preparation system (GV Instruments, Manchester, UK) at the Korea Basic Science Institute (KBSI). To determine oxygen isotopic composition, 0.25 mL water sample was transferred to a 5-mL Labco Exetainer® vial (Labco Ltd., High Wycombe, UK) and equilibrated with CO_2_ gas in a glove bag overnight at 25±0.1°C [3]. The CO_2_ gas was subsequently extracted and cryogenically purified. For hydrogen isotope analyses, metallic zinc was used to produce hydrogen gas using an automatic online sample preparation system (GV Instruments, Euro PyrOH, Manchester, UK).

To remove lipids and other contaminants, scalp hair samples were cleaned several times with acetone (HPLC grade, Merck, Germany) and ultrapure water (Pure Power II+ water purification system, Human Corporation, Seoul, Korea) according to the treatment procedure recommended by the International Atomic Energy Agency [4]. The samples were air-dried and cryogenically ground into a fine powder using a ball mill (Retsch, MM400, Haan, Germany). The hair samples were sent to the USGS to analyze oxygen and hydrogen isotopic compositions. The oxygen and hydrogen isotopic compositions of the hair samples were analyzed at least in duplicate using standard reference materials (USGS42 and 43) at the Stable Isotope Lab of the United States Geological Survey in Reston, Virginia.

All the isotopic ratios are reported in delta (δ) notation relative to Vienna Standard Mean Ocean Water (VSMOW), where δ (‰) = [(R_sample_/R_standard_) – 1] × 1000, and R = ^2^H/^1^H or ^18^O/^16^O. The analytical reproducibilities for δ^18^O and for δ^2^H were ±0.1‰ and ±1‰ for water samples, respectively, and 0.25‰ and below 2‰ for hair samples, respectively. Deuterium excess values were calculated following Dansgaard [2] as *d* = δ^2^H – (8 × δ^18^O).

### 2.6 Interpolated maps

Based on oxygen and hydrogen isotopic compositions, spatial patterns of water samples and human hair in South Korea were generated using ArcGIS v10.1 (ESRI, Redlands, CA, USA). The inverse distance weighted (IDW) interpolation method was used to analyze spatial patterns because IDW is intuitive and efficient (Azpurua and Ramos, 2010). For IDW, we used the default in the ArcGIS that included the inverse of the distance raised to the 2^nd^ power. Spatial interpolation was performed using IDW, which assigns values to unknown points based on a weighted average of the values at known points.

## Declaration of Competing Interest

The authors declare that they have no conflict of interest.
